# A zebrafish model of NEXMIF-related encephalopathy identified drivers of hyperlocomotion and provides a scalable platform for drug discovery

**DOI:** 10.3389/fcell.2026.1899363

**Published:** 2026-10-08

**Authors:** Beatriz Bernal-Bermúdez, Francisco J. Martínez-Morcillo, Inmaculada Fuentes-Martínez, Alicia Martínez-López, María L. Cayuela, Victoriano Mulero, Diana García-Moreno

**Affiliations:** 1 Departamento de Biología Celular e Histología, Facultad de Biología, Universidad de Murcia, Murcia, Spain; 2 Instituto Murciano de Investigación Biosanitaria (IMIB-Pascual Parrilla), Murcia, Spain; 3 Centro de Investigación Biomédica en Red de Enfermedades Raras (CIBERER), ISCIII, Madrid, Spain; 4 Hospital Clínico Universitario Virgen de la Arrixaca, Murcia, Spain

**Keywords:** chemical screening, neurodevelopmental disorder, NEXMIF, rare disease, zebrafish

## Abstract

X-linked Intellectual Disability type 98 (XLID-98) is a neurodevelopmental disorder with a broad phenotypic spectrum caused by pathogenic variants in the neurite extension and migration factor (*NEXMIF*) gene, formerly known as *KIAA2022, KIDLIA*, or *XPN* (OMIM 300524). To investigate the pathogenic mechanisms of XLID-98, a double knock-out zebrafish model (*nexmifa/b* ^−/−^) was generated using CRISPR/Cas9 technology. Mutant larvae displayed limited morphological abnormalities, the most notable being an increase in total body length. They also exhibited a significant hyperlocomotor phenotype in the visual-startle OFF assay, which was attenuated by treatment with sodium valproate (VPA) and topiramate, thereby supporting the pharmacological validity of the model. Transcriptomic analysis of larval heads at seven dpf revealed 414 differentially expressed genes enriched in mitogen-activated protein kinase (MAPK) signaling, cell adhesion, and sodium transport pathways. Functional analyses demonstrated that simultaneous CRISPR/Cas9-mediated disruption of the upregulated genes *entpd3* (ectonucleoside triphosphate diphosphohydrolase 3) and *ddx43* (DEAD-box helicase 43) exacerbated the hyperlocomotor phenotype, suggesting that these genes may participate in a compensatory response, whereas reduced *kbtbd7* (kelch repeat and BTB domain-containing protein 7) activity may contribute to phenotype severity. A targeted screen of 87 natural compounds identified kahweol acetate, veratridine, and rauwolscine as potent inducers of *entpd3*. However, despite robust transcriptional activation, rauwolscine failed to improve behavioral outcomes, suggesting that modulation of a single molecular target may be insufficient for phenotypic rescue. Collectively, these findings establish the first zebrafish model of NEXMIF-related encephalopathy, identify *entpd3*, *ddx43*, and *kbtbd7* as candidate modulators of this behavioral phenotype, and provide a scalable platform for targeted therapeutic discovery in XLID-98.

## Introduction

Neurodevelopmental disorders (NDDs) are a heterogeneous group of conditions that manifest during early development and affect cognitive, motor, language, and social functions. Many rare NDDs have a genetic origin and exhibit substantial phenotypic and genotypic variability, including a wide spectrum of intellectual disability (ID), autism spectrum disorder (ASD), and epilepsy (Srivastava et al., 2019). A subgroup of these disorders follows an X-linked pattern of inheritance, resulting in pronounced sex-specific differences in clinical presentation (Dibbens et al., 2008; Mignot et al., 2019). In female individuals, X-chromosome inactivation (XCI) is a process that silences one of the two X chromosomes to balance gene dosage, generating cellular mosaicism. When a pathogenic variant is present on one X chromosome, skewed XCI may lead to preferential expression of either the mutant or wild-type (WT) allele, thereby modulating disease penetrance and severity (Heyne et al., 2018; Migeon, 2006; Plenge et al., 2002; Stamberger et al., 2021). This mechanism of sex-modulated expressivity is evident in several X-linked NDDs, including Rett syndrome and various X-linked intellectual disabilities (XLIDs), such as XLID-98 (Anastasescu et al., 2025; Van Esch et al., 2005).

XLID-98 is a rare NDD caused by pathogenic variants in the *Neurite Extension and Migration Factor* (*NEXMIF*) gene (formerly known as *KIAA2022, KIDLIA or XPN*; OMIM 300524), located at Xq13.3 within a 192 kb genomic region (Anastasescu et al., 2025; Stekelenburg et al., 2022; Van Maldergem et al., 2013). *NEXMIF* encodes a large nuclear protein of 1,516 amino acids that is predominantly expressed in neurons during late embryonic and early postnatal development (Gilbert and Man, 2016; Magome et al., 2013). Although its precise molecular function remains incompletely defined, NEXMIF has been implicated in neuronal migration, axonal growth, and synaptic organization—processes essential for normal brain development (Stekelenburg et al., 2022; Cantagrel et al., 2004). Accordingly, pathogenic *NEXMIF* variants are associated with a broad clinical spectrum, including ASD, epilepsy with or without ID, microcephaly, strabismus, and postnatal growth delay, with disease severity often correlating with the degree of functional loss (Anastasescu et al., 2025; Chen et al., 2022; Sahajpal et al., 2024; Ye et al., 2024).

Epilepsy represents a prominent and highly variable feature of XLID-98 (Bernardo et al., 2024). Beyond XCI, a mechanism of cellular interference—previously described in *PCDH19*-related epilepsy—has been proposed to contribute to disease pathogenesis. In this model, mosaic co-existence of WT and mutant neurons disrupts N-cadherin-dependent cell-cell and cell-matrix interactions, processes in which NEXMIF is involved, thereby impairing neuronal network assembly and increasing seizure susceptibility (Dibbens et al., 2008; Magome et al., 2013; de Lange et al., 2016).

NEXMIF-related disorders arise from *de novo* or maternally inherited variants (Stamberger et al., 2021). The association between NEXMIF and ID was first established in two related male patients carrying a pericentric inversion of the X chromosome, Inv (X) (p22; p13.2) that disrupted both *KIAA2022* (now *NEXMIF*) and *P2YR8* (Cantagrel et al., 2004). The initial observation provided the first evidence linking NEXMIF to a neurodevelopmental disorder.

Since then, more than one hundred individuals with NEXMIF-related disorders have been reported, substantially broadening the mutational and clinical spectrum of XLID-98. Reported *NEXMIF* variants to date include missense, nonsense, and frameshift mutations, as well as deletions and duplications, distributed throughout the gene without a clear mutational hotspot, although exon 3—the largest coding exon—shows relative enrichment of lesions (Stamberger et al., 2021; Anastasescu et al., 2025). Importantly, most identified variants are predicted to result in loss of function, either through premature truncation or structural disruption leading to nonsense-mediated mRNA decay (NMD) (Stamberger et al., 2021; Brogna and Wen, 2009). In a cohort of 87 patients from 78 families, Stamberger et al. (2021) identified 58 unique NEXMIF variants, most of which were nonsense, frameshift, or large structural alterations expected to reduce or abolish protein expression (Stamberger et al., 2021).

Clinical manifestations of NEXMIF-related encephalopathy differ by sex. Affected male individuals typically present with more severe ID, marked language impairment, and behavioral abnormalities (Lambert et al., 2018; Lorenzo et al., 2018). In contrast, heterozygous female individuals more commonly exhibit epilepsy as the predominant clinical feature, often manifesting as absence seizures, myoclonic seizures, myoclonic absences, eyelid myoclonia, or phenotypes consistent with Jeavons syndrome (Panda et al., 2020; Alarcon-Martinez et al., 2019; Borlot et al., 2017). While early reports described asymptomatic female carriers, subsequent studies identified symptomatic female individuals with *de novo* truncating *NEXMIF* variants predicted to result in loss of function through NMD, mirroring the mechanism observed in affected male individuals (de Lange et al., 2016).

Seizures in many patients respond to antiseizure medications (ASMs) such as sodium valproate (VPA), topiramate (TPM), or lamotrigine. However, Ogasawara et al. (2020) reported that, despite its efficacy, VPA caused secondary amenorrhea in female patients, whereas replacement with clonazepam resulted in effective seizure control without adverse effects (Panda et al., 2020; Ogasawara et al., 2020). Despite partial therapeutic success, many individuals with NEXMIF-related encephalopathy develop drug-resistant epilepsy and persistent neurodevelopmental impairments, underscoring the need for improved disease models and targeted treatment strategies (Stamberger et al., 2021). Zebrafish is an established vertebrate model for neurodevelopmental disease modeling, behavioral assays for neuronal hyperexcitability, and drug discovery, owing to its genetic tractability and suitability for scalable pharmacological screening. Previous studies have demonstrated reduced locomotor hyperactivity following treatment with VPA or lamotrigine, supporting the predictive value of zebrafish behavioral assays for ASM screening (Berghmans et al., 2007; Afrikanova et al., 2013).

In this study, we generated a double knock-out zebrafish model (*nexmifa/b* ^−/−^) to investigate the molecular and functional mechanisms underlying NEXMIF-related encephalopathy and to identify candidate therapeutic compounds through a medium-scale natural compound screening.

## Results

### Expression profiling and generation of a double *nexmifa/b* knockout zebrafish model

To establish a zebrafish model of Nexmif deficiency and investigate the functional consequences of its loss *in vivo*, we examined the evolutionary conservation and expression of its zebrafish orthologs. In zebrafish, the orthologous locus is represented by *nexmifa* and *nexmifb* (Zheng et al., 2022). The human NEXMIF protein is composed of 1,516 amino acids (aa) and belongs to the Neurite Extension and Migration Factor family, characterized by the Domain of Unknown Function 4683 (DUF4683). Although the overall sequence identity between human NEXMIF and the zebrafish proteins is moderate, both Nexmifa (1,253 aa) and Nexmifb (1,230 aa) retain the characteristic NEXMIF-family region containing DUF4683 (Figure 1A). DUF4683 is a conserved domain present in NEXMIF/neurite extension and migration factor family proteins, although its precise molecular function remains incompletely defined. Pairwise protein alignment against human NEXMIF revealed 32.1% identity and 43.8% similarity for Nexmifa, and 38.4% identity and 53.3% similarity for Nexmifb (Figure 1B).

**FIGURE 1:**
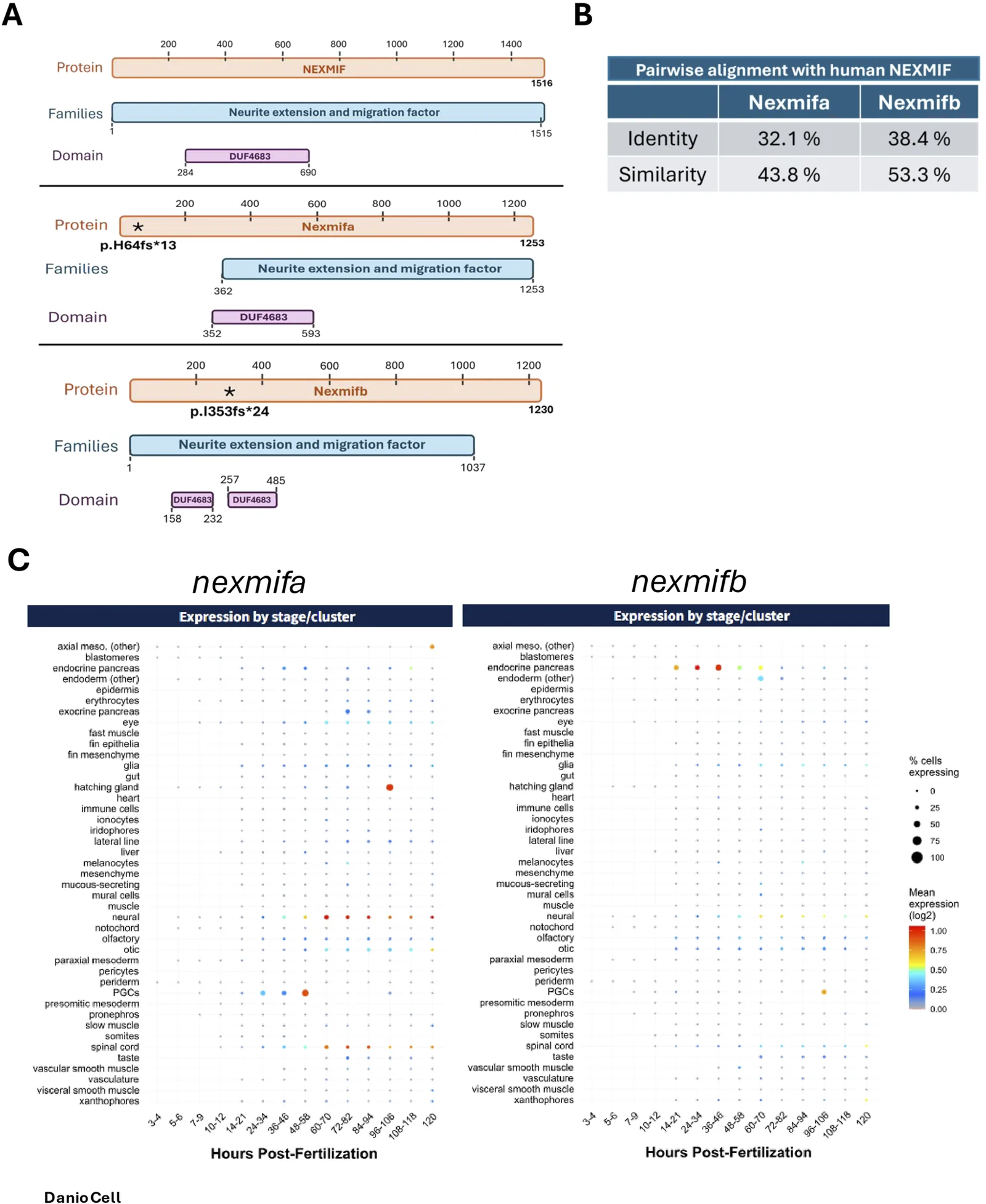
Evolutionary conservation and developmental expression of zebrafish *nexmifa* and *nexmifb* **(A)** Schematic representation of human NEXMIF and zebrafish Nexmifa and Nexmifb protein structure, showing the partial conservation of the NEXMIF-family/DUF4683 containing region. The positions of the frameshift mutations generated in the zebrafish paralogs are indicated with an asterisk **(B)** Pairwise amino acid sequence alignment showing the percentage of identity and similarity between human NEXMIF and the zebrafish paralogs Nexmifa and Nexmifb **(C)** DanioCell stage- and cluster-specific expression analysis of *nexmifa* and *nexmifb* during zebrafish development. Dot size represents the percentage of cells expressing each gene, and color intensity indicates mean expression level.

Analysis of expression patterns using DanioCell UMAP projections (https://daniocell.nichd.nih.gov) (Farrell et al., 2018; Sur et al., 2023) indicated that *nexmifa* and *nexmifb* share largely overlapping expression domains, with detectable expression in erythrocytes, mesenchyme, otic, and olfactory clusters, but showing their highest expression levels in neural, glial, and ocular cell groups (Supplementary Figure S1). Consistently, stage- and cluster-expression data further refined these patterns: *nexmifa* showed prominent expression in neural, otic, primordial germ cell (PGC), and spinal cord clusters, whereas *nexmifb* was most enriched in endocrine pancreas, glial, neural, olfactory, and otic populations (Figure 1C). Overall, *nexmifa* exhibited generally higher expression levels than *nexmifb*, yet both paralogs converge in key neurodevelopmental and sensory lineages, reinforcing their functional relevance in the zebrafish nervous system.

Based on the conservation of key NEXMIF-family features, their neural expression patterns, and the presence of two zebrafish *NEXMIF* orthologs, we generated a double loss-of-function model targeting both genes using CRISPR/Cas9 genome editing. Simultaneous disruption of *nexmifa* and *nexmifb* was selected to minimize potential functional redundancy and reduce overall Nexmif activity, thereby providing a robust zebrafish model of Nexmif deficiency. Gene-specific guide RNAs (gRNAs), whose sequences are listed in Supplementary Table S1, were microinjected into one-cell stage embryos, resulting in F0 mosaic founders carrying mutations in both genes. Gene-editing efficiency was assessed using the ICE tool from Synthego (https://ice.synthego.com/#/) (Conant et al., 2022), which provides a knockout score estimating the proportion of edited sequences carrying disruptive indels, particularly those that are not multiples of three and are therefore predicted to generate frameshifts, premature stop codons, and truncated proteins. The estimated knockout scores were 81% for *nexmifa* and 45% for *nexmifb* (Supplementary Figure S2A,B). These founders were raised to adulthood and crossed to produce the F1 generation. Genotyping confirmed successful germline transmission of the mutations, resulting in double heterozygous (*nexmifa/b* ^+/−^) offspring. These were further crossed to obtain the F2 generation, from which double homozygous mutants (*nexmifa/b* ^−/−^) were identified. From this point forward, these mutants are referred to as “*nexmifa/b*” mutants or “*nexmifa/b* ^−/−^” throughout the text.

Sanger sequencing confirmed frameshift-inducing deletions in both loci. In *nexmifa*, a 5-bp deletion resulted in a histidine-to-proline substitution at position 64 followed by a premature stop codon 13 aa downstream (p.H64fs*13) (Supplementary Figure S3A). In *nexmifb*, a 4-bp deletion produced a frameshift at position 353, introducing a premature stop codon 24 residues later (p.I353fs*24) (Supplementary Figure S3B). Both mutations are expected to disrupt the normal function of the respective proteins, supporting the generation of functional knockouts for both genes in the *nexmifa/b* mutant line (Figure 1A).

### *nexmifa/b* mutant zebrafish larvae exhibit increased body length with a preserved head-to-body ratio

Mutations in the *NEXMIF* gene, which are associated with XLID and ASD, have been studied in human patients using neuroimaging techniques such as magnetic resonance imaging (MRI). These studies frequently report either normal MRI results or mild abnormalities, such as increased extra-axial spaces in the frontal lobes or subtle cerebral atrophy, without consistent evidence of major structural brain defects (Stamberger et al., 2021; Stekelenburg et al., 2022; Alarcon-Martinez et al., 2019). Clinical evidence therefore suggests that *NEXMIF* variants rarely produce overt structural changes in the human brain. Guided by this observation, we examined zebrafish lacking Nexmifa/b to determine whether they display comparable macroscopic morphology.

To assess the impact of *nexmifa/b* loss on early development, we examined gross morphology, total body length, and head size of *nexmifa/b* ^−/−^ larvae at seven dpf (Figure 2A). Bright-field imaging revealed no overt external malformations compared with *nexmifa/b* ^+/+^ controls, but quantitative analysis showed that mutant larvae exhibited a significantly increased body length compared with controls (Figure 2B). Given that *NEXMIF* is associated with neurodevelopmental roles, we also assessed gross brain morphology by measuring the head size as a proxy for brain size. No significant differences in head size were found between genotypes (Figure 2C). To account for the increased body size, the head-to-body length ratio was calculated and found to be similar between *nexmifa/b* ^*+/+*^ and *nexmifa/b* ^−/−^ larvae (Figure 2D). Taken together, these findings indicate that, at this developmental stage, loss of *nexmifa/b* results in general growth alterations without detectable macroscopic effects on brain morphology.

**FIGURE 2:**
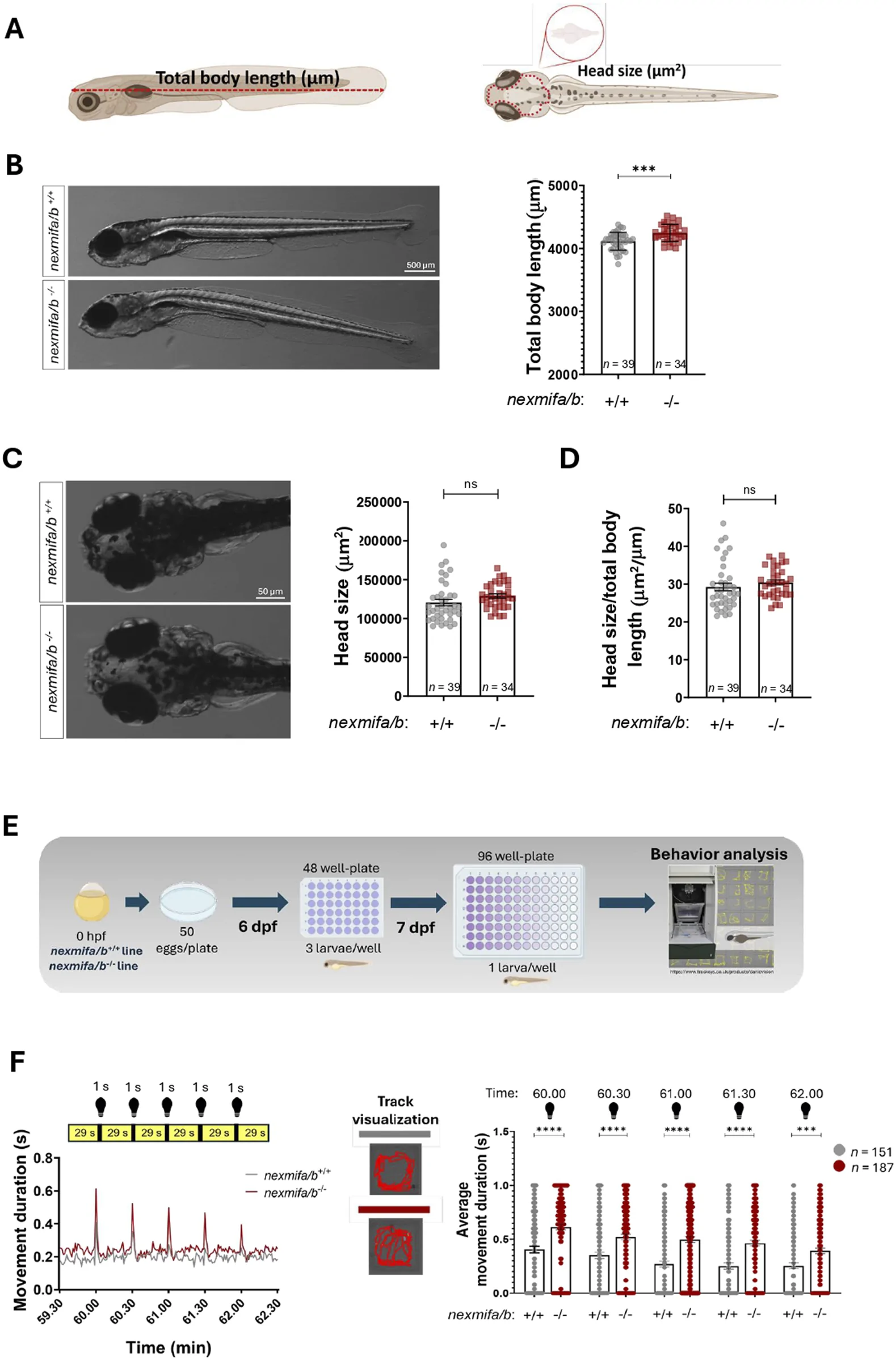
Loss of *nexmifa/b* increases body length and enhances locomotor responses in zebrafish larvae **(A)** Schematic representation of the morphological parameters measured in zebrafish larvae at seven dpf: total body length and head size **(B)** Representative images and quantification of body length **(C)** Representative images and quantification of head size **(D)** Quantification of the head size-to-total body length ratio **(E)** Experimental setup: three larvae were placed in a 48-well plate at six dpf, then individually transferred to a 96-well plate (one per well) for testing at seven dpf using the DanioVision system **(F)** Movement duration analysis during the final 3 min of the Visual-Startle OFF assay. Left, schematic representation of the five 1-s dark pulses separated by 29-s light intervals and time-course of movement duration in *nexmifa/b* ^*+/+*^ and *nexmifa/b* ^*−/−*^ larvae. Middle, representative swimming trajectories at the peak response to a dark pulse. Right, quantification of average movement duration at each dark-pulse peak. Each dot represents one individual larva; bars indicate mean ± SEM. *P*-values were calculated by Mann–Whitney U test. ***p ≤ 0.001; ****p ≤ 0.0001. Ns: non-significant. dpf: days post fertilization. min: minutes; s: seconds.

### Loss of *nexmifa/b* function enhances behavioral responses in zebrafish larvae

The *NEXMIF* gene plays a critical role in neurodevelopment, with mutations being linked to XLID and epilepsy (Webster et al., 2017). Zebrafish offer a powerful model system for studying NDDs due to their genetic conservation, optical transparency, and well-established behavioral assays (Kalueff et al., 2013). Our initial morphological analysis revealed growth alterations without detectable macroscopic effects on head morphology between *nexmifa/b* ^+/+^ and *nexmifa/b* mutant larvae, which aligns with the neurological and behavioral nature of NEXMIF-related disorders, where pronounced physical malformations are generally absent (Stamberger et al., 2021; Van Maldergem et al., 2013). Based on this observation, we hypothesized that *nexmifa/b* loss might enhance behavioral responses associated with neuronal hyperexcitability, a feature of several neurodevelopmental disorders, including developmental and epileptic encephalopathies. To test this hypothesis, we conducted locomotor activity assays using the DanioVision system (Figure 2E).

We employed the Visual-Startle OFF protocol, adapted from Weinschutz Mendes et al. (2023), which evaluates responses to dark pulses (Weinschutz Mendes et al., 2023). The Visual-Startle ON protocol was also tested but did not yield consistent genotype-dependent responses. The Visual-Startle OFF protocol consisted of a 60-min acclimation period under constant illumination, followed by five 1-s dark pulses, each separated by 29 s of light. No consistent genotype-dependent differences were detected during the 60-min acclimation period. Locomotor activity was quantified using DanioVision software, focusing on three parameters: movement duration, distance moved, and maximum acceleration. Analysis of the final 3 min of the recording—including the last 29 s of acclimation and the dark pulse period—revealed that *nexmifa/b* ^−/−^ larvae exhibited increased movement compared to *nexmifa/b* ^+/+^ controls during the dark pulses, a pattern also evident in representative swimming trajectories. Specifically, movement duration at the peak of each dark pulse was consistently and significantly elevated in mutant larvae across all five pulses (Figure 2F). Likewise, both distance moved and maximum acceleration (Supplementary Figure S4A,B) were significantly higher in *nexmifa/b* ^−/−^ larvae during dark pulses. These results suggest that the loss of *nexmifa/b* function leads to heightened behavioral responses to visual stimuli. This phenotype may reflect behavioral hyperlocomotion, consistent with the neurological manifestations, including epilepsy, commonly observed in individuals with NEXMIF-related disorders (Stamberger et al., 2021; Baraban et al., 2005).

### Effects of ASMs in *nexmifa/b* mutant zebrafish larvae

Following the characterization of the hyperlocomotor phenotype in *nexmifa/b* mutant zebrafish larvae, we investigated whether this altered behavior could be reversed through pharmacological treatment. Given that *NEXMIF* mutations are associated with epilepsy in humans (Stamberger et al., 2021), we tested two widely used ASMs—VPA and TPM—both of which are clinically prescribed for seizure control in patients with NEXMIF-related disorders. These compounds have also demonstrated efficacy in zebrafish seizure-related assays, where they reduce hyperlocomotor or seizure-related behavioral activity (Berghmans et al., 2007; Patton et al., 2021).

To establish effective treatment conditions, we first performed preliminary concentration–response assays for both drugs, guided by previous studies in zebrafish seizure-related assays (Berghmans et al., 2007; Afrikanova et al., 2013; Gawel et al., 2020). Based on the reproducibility of the responses, we selected 2.5 mM VPA and 5 μM TPM as working concentrations in all subsequent assays. To perform these experiments, *nexmifa/b* mutant larvae at six dpf were exposed to VPA or TPM by bath immersion for 24 h (Figure 3A). VPA- and TPM-treated *nexmifa/b* mutant larvae exhibited a significant reduction in movement duration during the dark pulse periods compared to vehicle-treated mutants. Movement track visualizations further supported these findings, with VPA- and TPM-treated larvae displaying shorter and more regulated swimming patterns relative to untreated mutants. This reduction was consistent across all dark pulses (Figures 3B,C). Likewise, both distance moved and maximum acceleration were significantly reduced in the treated groups (Supplementary Figure S5A-D).

**FIGURE 3:**
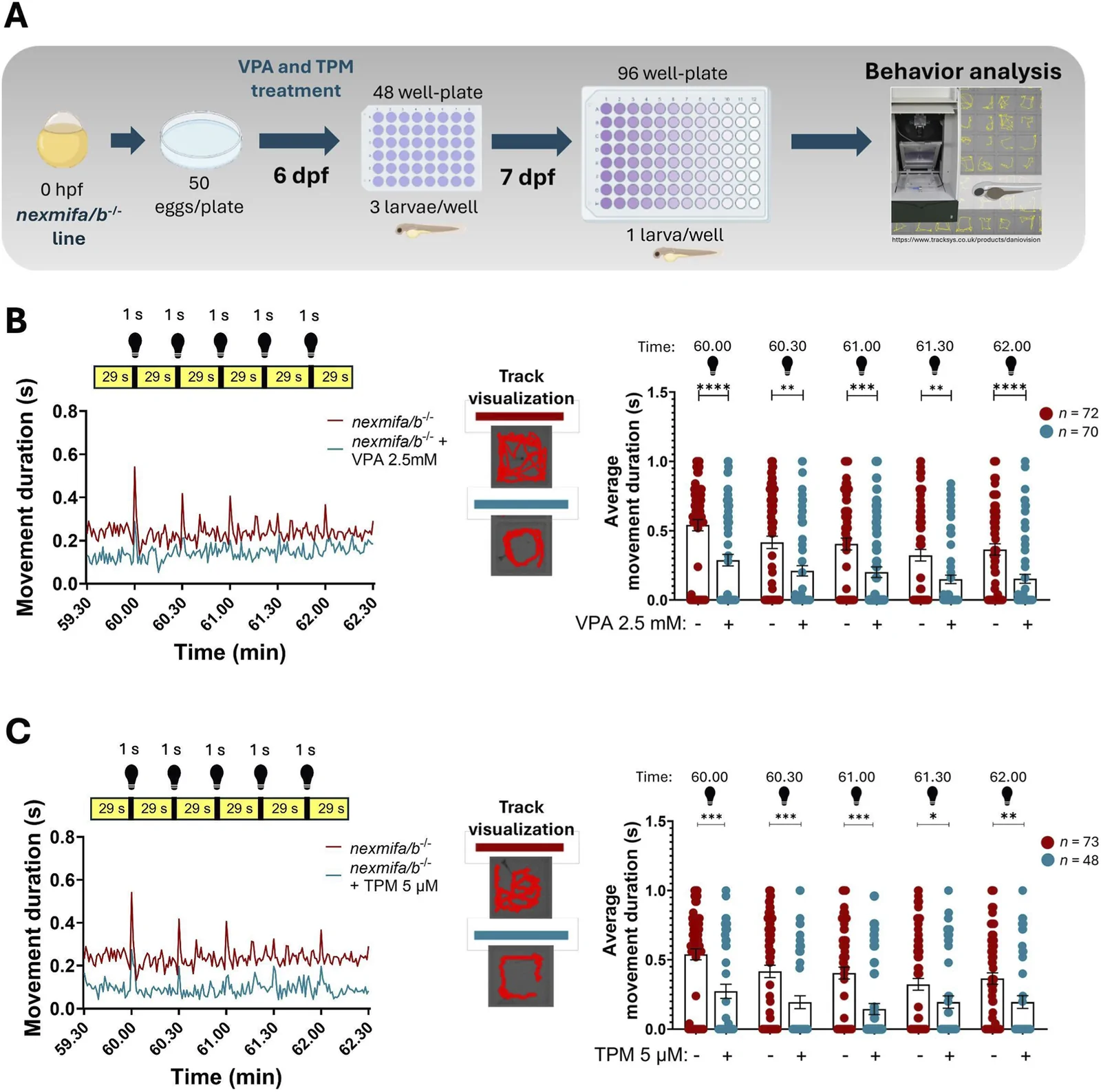
VPA and TPM attenuate the hyperlocomotor phenotype in *nexmifa/b* mutant larvae **(A)** Experimental setup: three larvae were placed in each well in a 48-well plate and treated with 2.5 mM VPA, 5 μM TPM, or DMSO (control) at six dpf; then, individual larvae were transferred to a 96-well plate (one per well) for testing at seven dpf using the DanioVision system **(B)** Movement duration analysis in VPA-treated larvae during the final 3 min of the Visual-Startle OFF assay. Left, schematic representation of the five 1-s dark pulses separated by 29-s light intervals and time-course of movement duration in both groups. Middle, representative swimming trajectories at the peak response to a dark pulse. Right, quantification of average movement duration at each dark-pulse peak **(C)** Movement duration analysis in TPM-treated larvae during the final 3 min of the Visual-Startle OFF assay. Left, schematic representation of the five 1-s dark pulses separated by 29-s light intervals and time-course of movement duration in both groups. Middle, representative swimming trajectories at the peak response to a dark pulse. Right, quantification of average movement duration at each dark-pulse peak. Each dot represents one individual larva; bars indicate mean ± SEM. VPA, *n* = 72 for untreated, *n* = 70 for VPA-treated; TPM *n* = 73 for untreated, *n* = 48 for TPM-treated, *P*-values were calculated by Mann–Whitney U test. *p ≤ 0.05; **p ≤ 0.01; ***p ≤ 0.001; ****p ≤ 0.0001. DMSO, dimethyl sulfoxide; dpf: days post fertilization; min: minutes; s: seconds.

Taken together, these pharmacological experiments demonstrate that both VPA and TPM significantly attenuate hyperlocomotor behavior in *nexmifa/b* mutant larvae.

### Transcriptomic analysis identifies molecular changes associated with nexmifa/b deficiency

To investigate the molecular pathways disrupted in our model and to identify potential therapeutic targets for NEXMIF-related NDDs, we performed RNA sequencing (RNA-seq) on heads from seven dpf *nexmifa/b* ^+/+^ and *nexmifa/b* ^−/−^ zebrafish larvae. Principal component analysis (PCA) showed a clear separation between *nexmifa/b* ^*+/+*^ and *nexmifa/b* ^*−/−*^ samples, while biological replicates clustered closely within each genotype, indicating good reproducibility of the transcriptomic data (Figure 4A). The analysis revealed 414 DEGs, with 166 upregulated and 248 downregulated in mutants compared with controls (Figure 4B). Among the DEGs identified in *nexmifa/b* mutant larvae, we prioritized *entpd3, ddx43*, and *kbtbd7* for further analysis based on a combination of statistical significance, direction of differential expression, biological relevance to neuronal processes, robust validation by RT-qPCR, and suitability for rapid functional testing in zebrafish larvae. Representative DEGs are shown in the heatmap (Figure 4C), including the upregulated genes *entpd3* and *ddx43* and the downregulated gene *kbtbd7,* which were selected for subsequent functional analyses.

**FIGURE 4:**
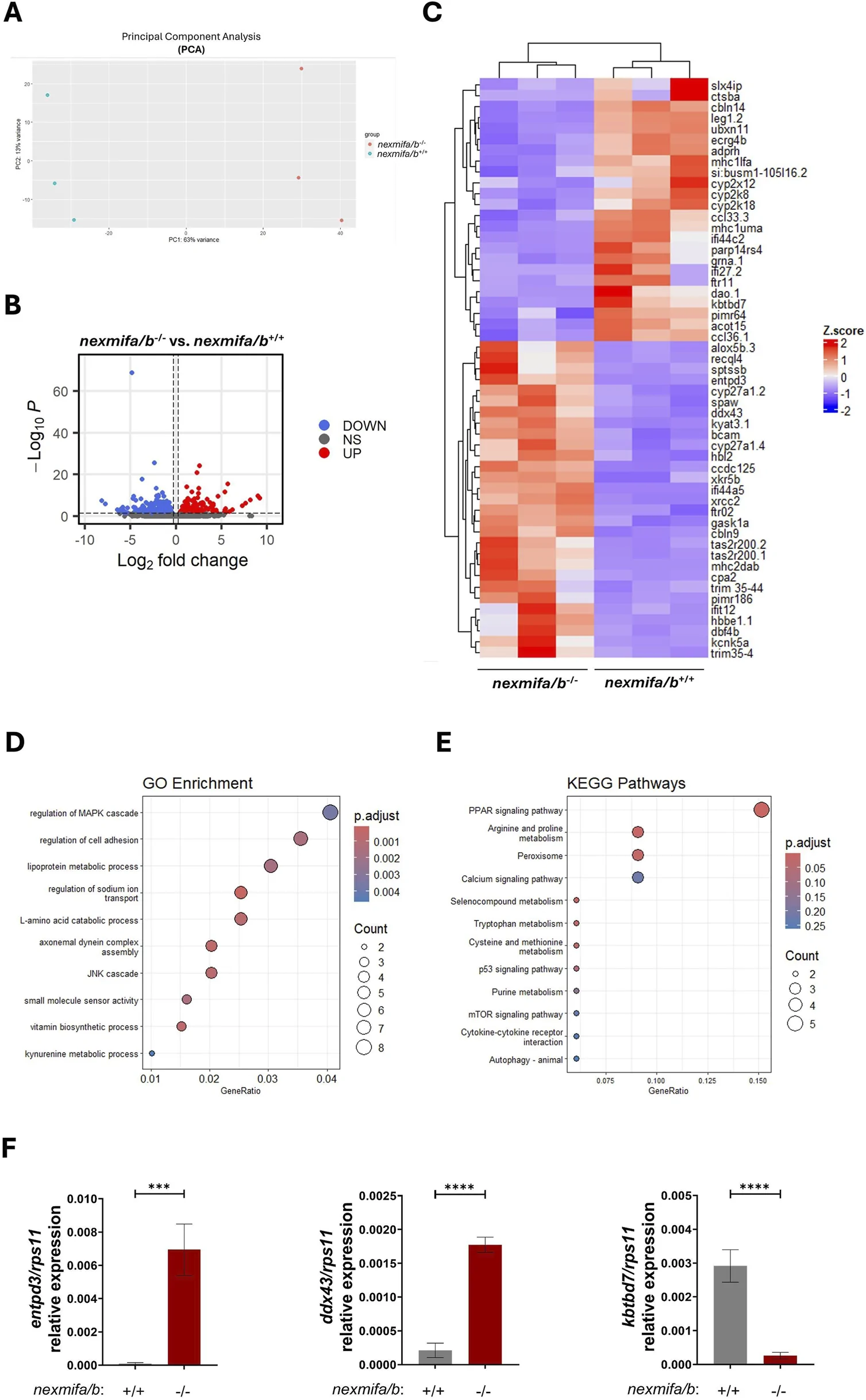
Transcriptomic analysis reveals differentially expressed genes (DEGs) in the heads of *nexmifa/b* ^*+/+*^ and *nexmifa/b* ^*−/−*^ larvae at seven dpf **(A)** Principal component analysis (PCA) of RNA-seq samples from *nexmifa/b^+/+^* and *nexmifa/b^−/−^* larvae. Each point represents one biological replicate **(B)** Volcano plot of 414 DEGs with log_2_ fold change ≥0.25 or ≤ −0.25. Red and blue dots represent upregulated and downregulated genes, respectively **(C)** Heatmap of the top DEGs (log_2_ fold change ≥1.5 or ≤ −1.5) in both groups. Each condition includes three independent biological replicates **(D,E)** Dot plots representing the most significantly enriched pathways based on GO and KEGG pathways. Dot size indicates the number of DEGs involved in each pathway; color indicates statistical significance based on adjusted *p*-values **(F)** Validation of selected genes *entpd3*, *ddx43*, and *kbtbd7* from *nexmifa/b* ^*+/+*^ and *nexmifa/b* ^*−/−*^ larval heads. Each dot represents a biological replicate. *P*-values were calculated using Student’s t-test. ***p ≤ 0.001; ****p ≤ 0.0001. dpf: days post fertilization.

Gene Ontology (GO) enrichment analysis identified several biological processes relevant to neurological function, including regulation of the MAPK cascade, cell adhesion, and sodium ion transport (Figure 4D). Additionally, Kyoto Encyclopedia of Genes and Genomes (KEGG) pathway analysis revealed enrichment in pathways related to metabolism, calcium signaling, and peroxisome proliferator-activated receptors (PPAR) signaling, among others (Figure 4E).

Among the upregulated genes identified in *nexmifa/b* mutant larvae were *entpd3* and *ddx43*, both of which may play functionally relevant roles in the altered neurodevelopmental phenotype. The *ENTPD3* gene encodes an ectonucleotidase involved in the degradation of extracellular ATP, which is critical for purinergic signaling, a key mechanism in neuron-glia communication and synaptic regulation (Vongtau et al., 2011). In zebrafish, *entpd3* expression has been reported in sensory and hypothalamic neurons, suggesting a potential role in modulating neural circuits (Appelbaum et al., 2007). *DDX43*, a member of the DEAD-box RNA helicase family, participates in various aspects of RNA metabolism, including splicing, transport, and translation. Although its specific function in the nervous system remains poorly understood, altered *DDX43* expression has been linked to neuropsychiatric disorders, indicating its possible involvement in neural gene regulation or RNA processing during brain development (Wockner et al., 2015). In addition, among the downregulated genes identified in *nexmifa/b* mutant larvae was *kbtbd7* (*Kelch repeat and BTB domain-containing 7*), an E3 ubiquitin ligase implicated in various pathological processes. Recent findings by Shen et al. (2024) associate *KBTBD7* with the regulation of neuroinflammation and neuronal damage in the context of septic brain injury (Shen et al., 2024).

To validate the RNA-seq findings, we performed RT-qPCR for *entpd3*, *ddx43* and *kbtbd7* using RNA from the same biological samples analyzed by RNA-seq. The RT-qPCR results confirmed the differential expression patterns observed in the transcriptomic data (Figure 4F).

### CRISPR/Cas9-mediated targeting of *entpd3*, *ddx43*, and *kbtbd7* affects behavior in *nexmifa/b* mutant larvae

Given the differential mRNA levels of *entpd3*, *ddx43*, and *kbtbd7* in *nexmifa/b* mutant larvae, we hypothesized that these genes may either participate in compensatory responses or contribute to phenotype severity. To assess their functional relevance, we performed targeted F0 CRISPR/Cas9-mediated gene disruption of each gene in the *nexmifa/b* mutant background and evaluated the resulting effects on locomotor behavior.

As a first step, we used the CRISPR-Cas9 system to target *entpd3* or *ddx43* in *nexmifa/b* mutant larvae. Deconvolution analysis using the ICE tool from Synthego (https://ice.synthego.com/#/) (Conant et al., 2022) demonstrated high editing efficiencies for both genes, with indel rates/knockout scores of 97%/83% for *entpd3* (Supplementary Figure S2C) and 94%/72% for *ddx43* (Supplementary Figure S2D). To evaluate the impact of editing *entpd3* and *ddx43* on locomotor activity, we employed the Visual-Startle OFF protocol in the DanioVision system, consistent with previous experiments (Figure 5A).

**FIGURE 5:**
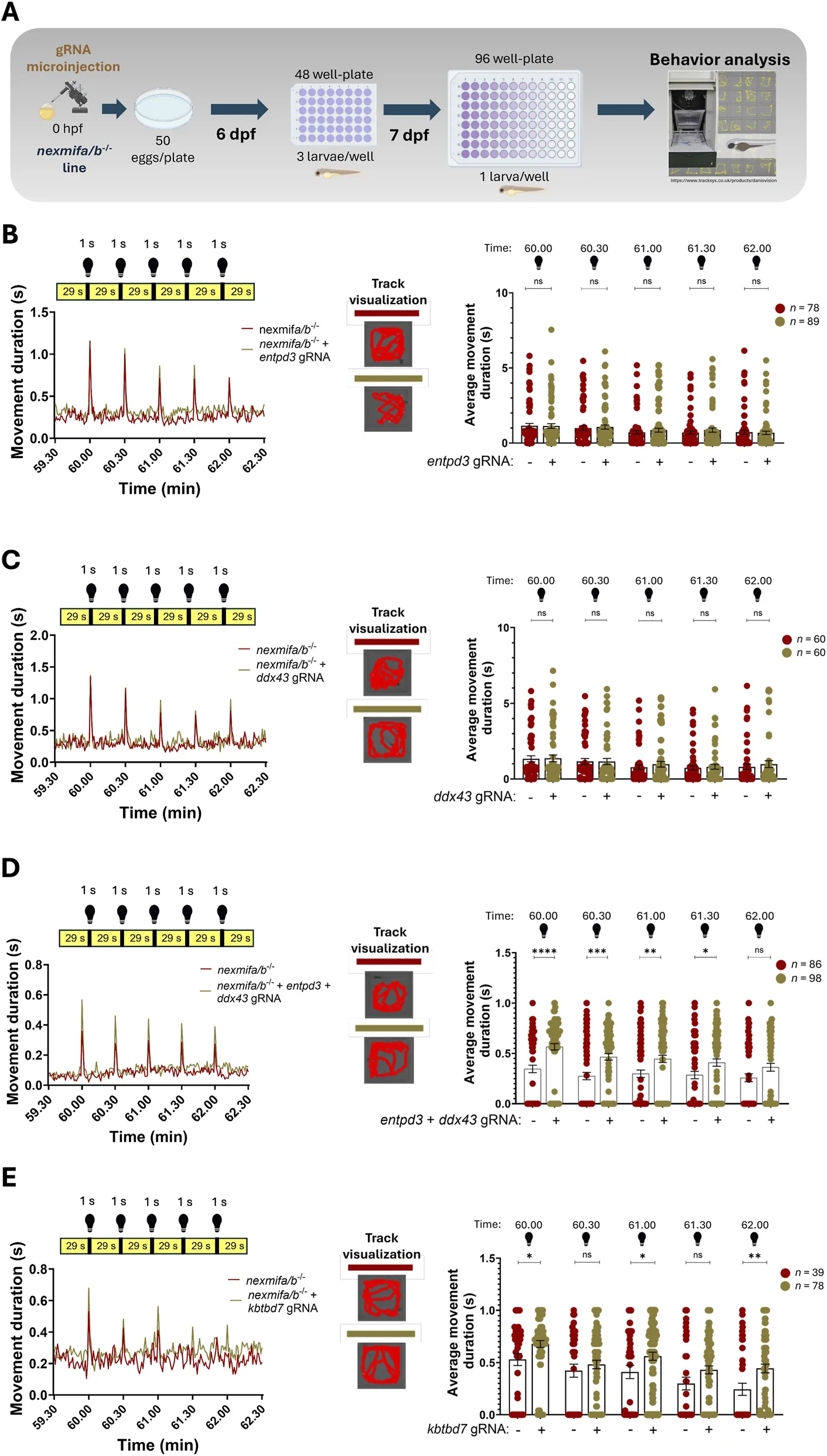
Effects of *entpd3*, *ddx43*, and *kbtbd7* gene targeting on behavioral phenotypes in *nexmifa/b* mutants **(A)** Experimental design illustrates the process from microinjection of entpd3, *ddx43, entpd3* + *ddx43*, or *kbtbd7* gRNA in the presence of recombinant Cas9. Three larvae were placed in a 48-well plate at six dpf, then individually transferred to a 96-well plate (one per well) for testing at seven dpf using the DanioVision system **(B–E)** Movement duration analysis during the final 3 min of the Visual-Startle OFF assay in *nexmifa/b* mutant larvae following targeting of *entpd3* **(B)**, *ddx43* **(C)**, *entpd3* + *ddx43* **(D)**, or *kbtbd7* **(E)**. Left, schematic representation of the five 1-s dark pulses separated by 29-s light intervals and time-course of movement duration in both groups. Middle, representative swimming trajectories at the peak response to a dark pulse. Right, quantification of average movement duration at each dark-pulse peak. Each dot represents one individual larva; bars indicate mean ± SEM. *entpd3* gRNA, *n* = 78 for *nexmifa/b* ^*−/−*^, *n = 89* for *entpd3* gRNA in *nexmifa/b* ^*−/−*^ larvae, *ddx43* gRNA, *n* = 60 for *nexmifa/b* ^*−/−*^, *n = 60 for ddx43* gRNA in *nexmifa/b* ^*−/−*^ larvae, *n* = 86 for *nexmifa/b* ^*−/−*^, *n* = 98 for *entpd3* + *ddx43* gRNA in *nexmifa/b* ^*−/−*^ larvae, *n* = 39 for *nexmifa/b* ^*−/−*^, *n* = 78 for *kbtbd7* gRNA in *nexmifa/b* ^*−/−*^ larvae. *P*-values were calculated by Mann–Whitney U test. Ns: non-significant. *p ≤ 0.05; **p ≤ 0.01; ***p ≤ 0.001; ****p ≤ 0.0001. dpf: days post fertilization; min: minutes; s: seconds.

Locomotor parameters—including movement duration, distance moved, and maximum acceleration—were quantified using DanioVision software. Unexpectedly, *entpd3*- or *ddx43*-targeted *nexmifa/b* mutants exhibited locomotor activity comparable to those injected with STD control gRNA; no significant differences were observed in movement duration, a pattern also evident in representative swimming trajectories (Figures 5B,C). Likewise, no significant differences were observed in distance moved or maximum acceleration between *entpd3*- and *ddx43*-targeted larvae and control mutants (Supplementary Figure S6A-D). Taken together, these findings suggest that individual targeting of *entpd3* or *ddx43* is insufficient to modify the hyperactive locomotor phenotype in *nexmifa/b* mutants. This supports the notion that additional or synergistic mechanisms may contribute to the observed behavioral abnormalities.

We next explored whether simultaneous disruption of both genes might be required, or whether other compensatory molecular mechanisms continued to mask potential effects. Following dual gene disruption of *entpd3* and *ddx43*, *nexmifa/b* mutant larvae exhibited a consistent increase in movement duration at the peak of four consecutive dark pulses compared with larvae injected with STD control gRNA. These findings were further supported by locomotor track visualizations, which showed more active and extended movement patterns in the dual-targeted group (Figure 5D). Similarly, both distance moved and maximum acceleration were significantly elevated in the co-injected group (Supplementary Figure S7A,B).

In summary, while individual targeting of *entpd3* or *ddx43* did not significantly alter locomotor behavior, their combined targeting exacerbated the hyperactive phenotype, suggesting that these genes may participate in a compensatory response in the context of *nexmifa/b* mutation.

Then, to explore the potential role of *kbtbd7* in the *nexmifa/b* loss-of-function phenotype, we applied the same F0 CRISPR/Cas9-mediated gene disruption strategy used for *entpd3* and *ddx43* (Figure 5A). Editing efficiency for the *kbtbd7* gRNA was lower than that achieved for *entpd3* and *ddx43*, with an indel rate/knockout score of 26%/26% (Supplementary Figure S2E). As with *entpd3* and *ddx43*, this knockout score indicates partial and mosaic genetic disruption rather than complete loss of function.

Following genetic perturbation of *kbtbd7*, movement duration at the peak of each dark pulse was consistently and significantly increased compared to *nexmifa/b* ^−/−^ larvae injected with STD gRNA, and visual analysis of locomotor traces confirmed these findings (Figure 5E). Similarly, both distance moved and maximum acceleration were significantly elevated following genetic perturbation of kbtbd*7* (Supplementary Figure S8A,B). These results suggest that the reduced *kbtbd7* transcript levels may contribute to the hyperlocomotor phenotype and support kbtbd7 as a candidate modifier of phenotype severity in the context of *nexmifa/b* mutation. Unlike *entpd3* and *ddx43*, whose inhibition appeared to disrupt compensatory responses and worsen the phenotype only when combined, additional targeting of *kbtbd7*—a gene already downregulated in the mutants—resulted in a further increase in locomotor activity. Overall, the behavioral changes observed following F0 CRISPR/Cas9-mediated targeting of *entpd3, ddx43*, and *kbtbd7* are interpreted as functional evidence supporting the involvement of these genes in modulating the hyperlocomotor phenotype, while avoiding overstatement regarding complete gene ablation or definitive molecular mechanism.

### Screening of Natural Product Library in *nexmifa/b* zebrafish model

After establishing the *nexmifa/b* zebrafish model and identifying molecular pathways disrupted by Nexmifa/b deficiency, we performed a medium-scale chemical screen. For this purpose, we utilized the SCREEN-WELL® Natural Product Library (Enzo Life Sciences), a curated collection of over 500 structurally diverse, bioactive compounds. This library includes a wide array of natural metabolites—such as alkaloids, terpenes, and flavonoids—many of which possess neuroactive properties and have a long-standing history of use in traditional medicine.

Before initiating the screen, we confirmed by conventional RT-qPCR that transcript levels of *entpd3* in whole 3-dpf larvae reflected the upregulation previously observed in RNA-seq data from dissected larval heads (Figure 6A). To analyze the results of the screening, total RNA was extracted, and gene expression was assessed by one-step RT-qPCR, with *entpd3* selected as the primary molecular marker (Figure 6B).

**FIGURE 6:**
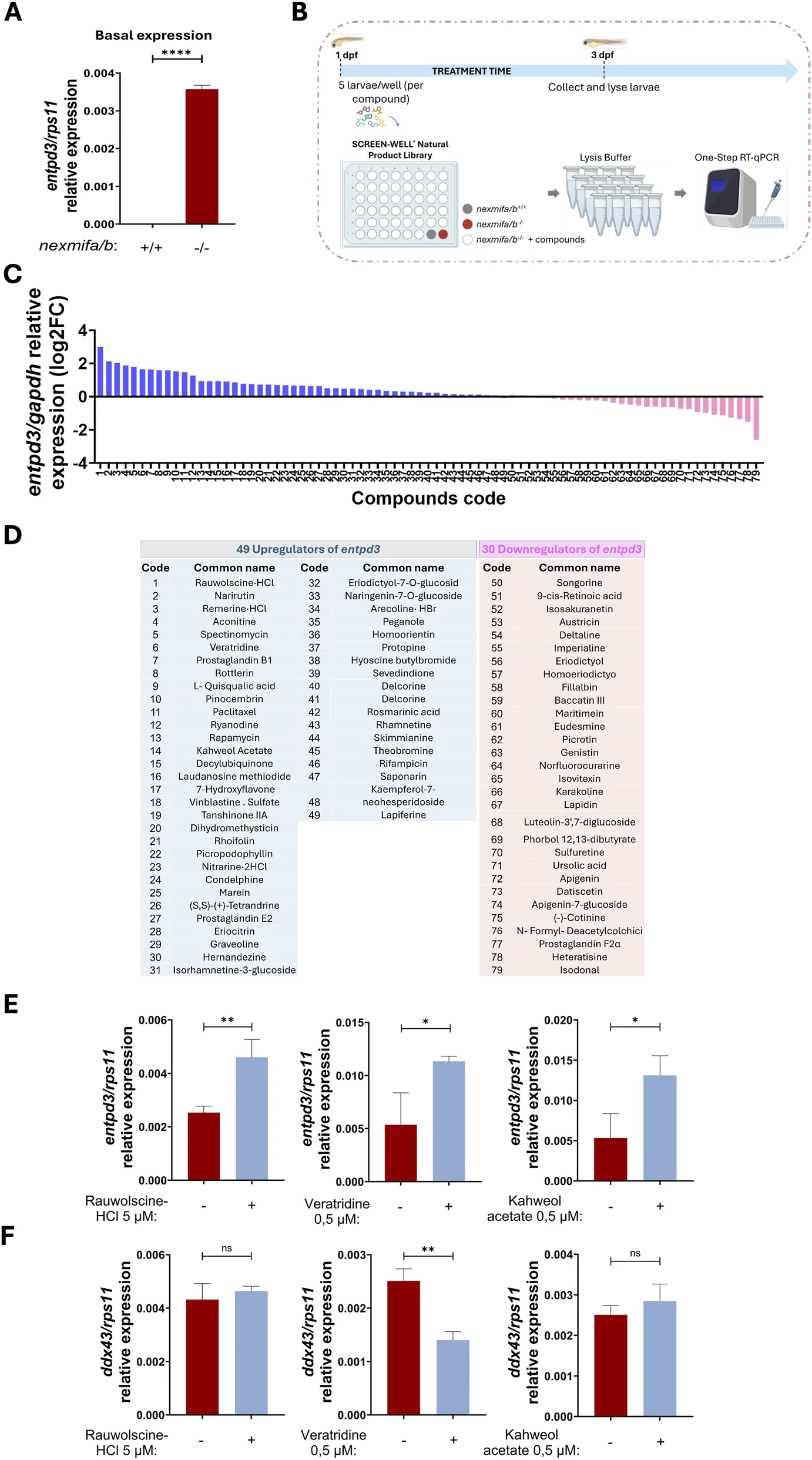
Natural product screen using transcript levels of *entpd3* as a molecular marker in the *nexmifa/b* mutant model **(A)** Validation of baseline mRNA levels of *entpd3* in whole larvae at three dpf by conventional RT-qPCR, prior to screening **(B)** Schematic representation of the natural product screen workflow: larvae were dechorionated at one dpf, exposed to individual compounds from the SCREEN-WELL® Natural Product Library for 48 h, followed by lysis, RNA extraction, and gene expression analysis by One-Step RT-qPCR **(C)** Distribution of 79 compounds based on *entpd3* mRNA levels. Gene expression is represented as log_2_ fold change (log_2_FC) relative to baseline levels in untreated *nexmifa/b* ^*−/−*^ larvae. Blue bars indicate compounds that induced upregulation of *entpd3*; pink bars indicate compounds that caused downregulation **(D)** Reference table linking compound codes to common names **(E)** Validation of the natural product screen identifies rauwolscine-HCl, veratridine, and kahweol acetate as inducers of *entpd3* transcript levels in *nexmifa/b* mutant model **(F)** *ddx43* mRNA levels in *nexmifa/b* ^*−/−*^ larvae treated with rauwolscine-HCl, veratridine, and kahweol acetate compared to DMSO-treated mutants. *P*-values were calculated using Student’s t-test. *p ≤ 0.05; **p ≤ 0.01; ****p ≤ 0.0001. Ns: non-significant. dpf: days post fertilization.

A total of 87 compounds were tested using the commercial library, in which compounds were supplied at a stock concentration of 2 mg/mL. Since the compounds have different molecular weights, this fixed mass concentration corresponded to different molar concentrations across the library. Of these, eight compounds were toxic, resulting in complete larval mortality during the 48-h exposure. The remaining 79 non-toxic compounds were included in the gene expression analysis (Figure 6C). Among these, 49 compounds (blue bars) further upregulated *entpd3* transcript levels, while 30 compounds (pink bars) led to downregulation relative to baseline levels in untreated *nexmifa/b* mutant larvae. A comprehensive reference table linking each compound code to its corresponding common name is provided in Figure 6D.

To select compounds for secondary validation, we focused on those that significantly modulated *entpd3* transcription, based on prior findings suggesting that *entpd3* upregulation in *nexmifa/b* ^−/−^ larvae may represent an endogenous compensatory response—potentially in combination with *ddx43—*to Nexmif deficiency. Among these, rauwolscine, veratridine and kahweol acetate were selected. Although the compounds in the commercial library were supplied at a fixed stock concentration of 2 mg/mL, the final molar concentration used in the primary screen for rauwolscine, veratridine and kahweol acetate was approximately 5 μM based on their molecular weights. For secondary validation, the compounds were repurchased individually and initially tested at this equivalent molar concentration. While rauwolscine was well tolerated at 5 μM, kahweol acetate and veratridine showed toxicity. Therefore, the concentration of these two compounds was reduced to 0.5 µM for all subsequent validation assays. After 48 h of treatment, larvae were collected at three dpf, and total RNA was extracted. Gene expression analysis via RT-qPCR confirmed that all three compounds significantly increased transcript levels of *entpd3* in *nexmifa/b* mutant larvae compared to vehicle-treated mutants (Figure 6E). Among them, rauwolscine elicited the most robust transcriptional response.

Given that simultaneous modulation of *entpd3* and *ddx43* was previously shown to be necessary for altering the behavioral phenotypes in *nexmifa/b* ^−/−^ larvae, we next evaluated whether treatment with these compounds also affected *ddx43* mRNA levels. RT-qPCR analysis showed that neither kahweol acetate nor rauwolscine significantly altered *ddx43* transcript levels. In contrast, veratridine—while upregulating *entpd3*—led to a significant downregulation of *ddx43* (Figure 6F). These results indicate that the transcriptional effects of the compounds are gene-specific, with each compound triggering distinct molecular responses.

Since rauwolscine induced the strongest upregulation of *entpd3* among the compounds tested, we next evaluated its potential to ameliorate the behavioral phenotype associated with Nexmif deficiency (Figure 7A). Quantitative analysis of locomotor parameters revealed no significant differences between rauwolscine–treated larvae and vehicle-treated *nexmifa/b* ^*−/−*^. Specifically, movement duration during dark pulse periods was comparable between groups—a pattern also evident in representative swimming trajectories (Figure 7B). Likewise, no significant differences were observed in both distance moved and maximum acceleration between rauwolscine-HCl–treated larvae and vehicle-treated *nexmifa/b* ^*−/−*^ (Supplementary Figure S9A,B).

**FIGURE 7:**
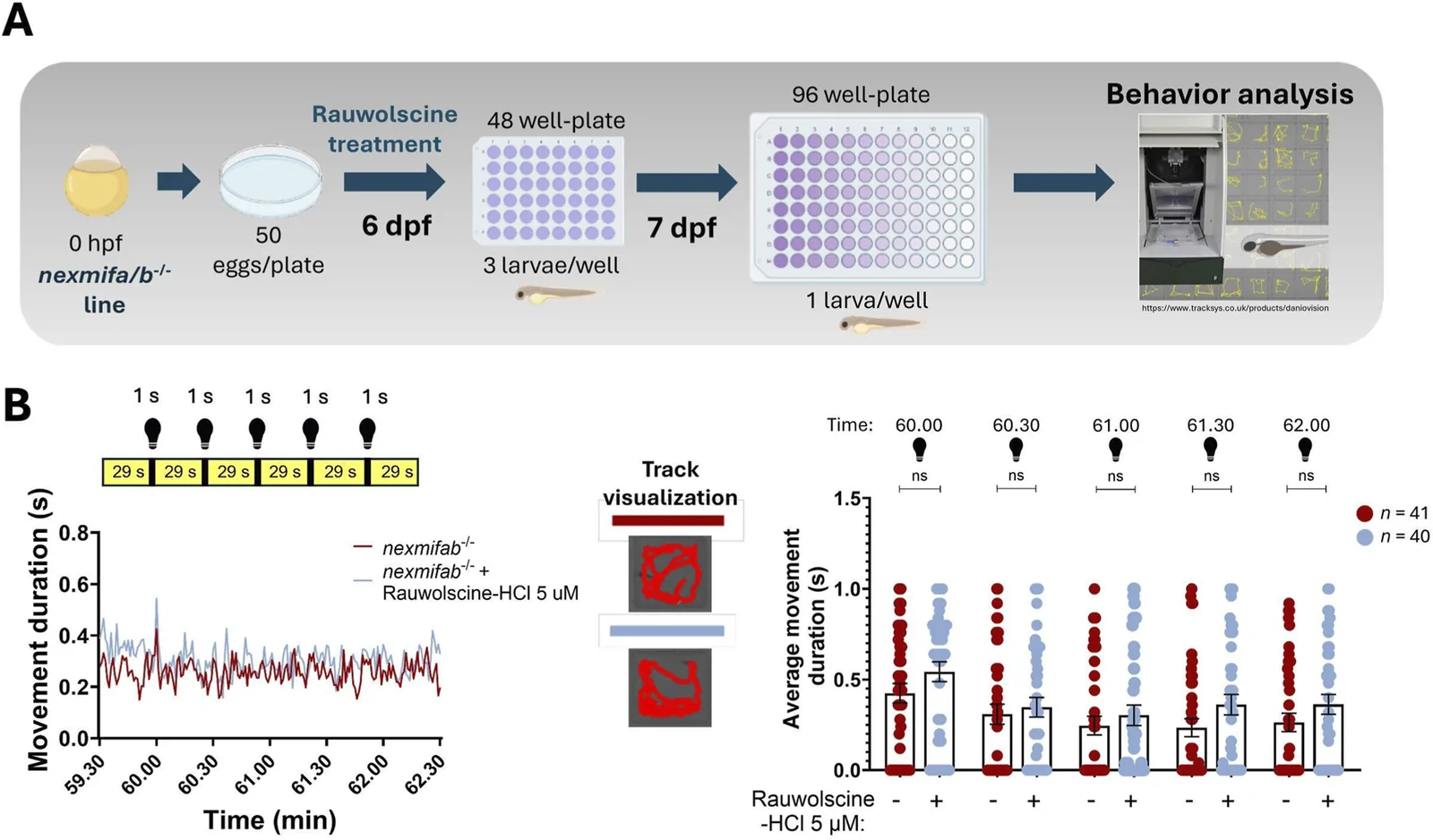
Behavioral phenotype is not rescued by rauwolscine-HCl treatment in the *nexmifa/b* mutant model **(A)** Experimental setup: three larvae were placed in each well in a 48-well plate and treated with 5 μM rauwolscine-HCl or DMSO (control) at six dpf, then individually transferred to a 96-well plate (one per well) for testing at seven dpf using the DanioVision system **(B)** Movement duration analysis in rauwolscine-HCl-treated larvae during the final 3 min of the Visual-Startle OFF assay. Left, schematic representation of the five 1-s dark pulses separated by 29-s light intervals and time-course of movement duration in both groups. Middle, representative swimming trajectories at the peak response to a dark pulse. Right, quantification of average movement duration at each dark-pulse peak. Each dot represents one individual larva; bars indicate mean ± SEM. *n* = 41 for untreated, *n* = 40 rauwolscine-HCl-treated *nexmifa/b* ^*−/−*^ larvae. *P*-values were calculated by Mann–Whitney U test. Ns: non-significant. dpf: days post fertilization; min: minutes; s: seconds.

These behavioral findings are consistent with the molecular data; while rauwolscine selectively increased mRNA levels of *entpd3*, it did not affect *ddx43* levels. This suggests that modulation of *entpd3* alone is insufficient to restore the locomotor phenotype in *nexmifa/b* mutant larvae. The lack of behavioral improvement despite robust transcriptional activation underscores the complexity of the disease mechanism and highlights the need for multi-target strategies in therapeutic development.

## Discussion

XLID-98 is one of approximately 7,000 rare diseases that collectively affect 3.5%–5.9% of the global population (Nguengang Wakap et al., 2020) yet still lack vertebrate models for mechanism-driven drug discovery. Most functional work on NEXMIF has relied on *in vitro* neurite-outgrowth assays or on murine models employing hemizygous males or partial/tissue-specific knockouts, as complete Nexmif-knockout males are infertile and homozygous females cannot be generated, while heterozygous females show variable social and cognitive phenotypes, leaving the systemic consequences of full gene loss largely unexplored. An important difference between the zebrafish and mouse models concerns reproductive phenotypes. Whereas complete Nexmif knockout male mice are infertile, such a phenotype has not been observed in our zebrafish model. This discrepancy may reflect species-specific reproductive biology, divergence between the duplicated zebrafish orthologs and mammalian NEXMIF, or differences in the physiological functions of NEXMIF across vertebrates (Van Maldergem et al., 2013; Mount et al., 2023). Our double-knockout zebrafish line therefore fills this gap by providing (1) early-life viability, (2) a quantifiable hyperlocomotor phenotype associated with neuronal hyperexcitability, and (3) scalability for drug screening—three features aligned with priorities promoted by the International Rare Diseases Research Consortium (IRDiRC), including scalable disease models and therapeutic discovery platforms (Boycott et al., 2019).

Photosensitive hyperlocomotion in the visual-startle OFF assay resembles the stimulus-induced behavioral abnormalities commonly observed in DEEs and mirrors phenotypes reported in established zebrafish models of Dravet syndrome and STXBP1-related encephalopathy (Grone et al., 2016; Whyte-Fagundes et al., 2026; Baraban et al., 2013). Crucially, this phenotype was significantly attenuated by VPA and TPM—first-line treatments that, together with lamotrigine and levetiracetam, achieve partial seizure control in many XLID-98 patients (Stamberger et al., 2021). The shared responsiveness to ASMs further supports the predictive value of this behavioral assay (Berghmans et al., 2007; Patton et al., 2021) and distinguishes our zebrafish model from existing mouse lines, in which pharmacological responsiveness has yet to be systematically investigated. The absence of macroscopic brain malformations in larvae aligns with the largely normal MRI findings in most affected individuals (Stekelenburg et al., 2022; Alarcon-Martinez et al., 2019), reinforcing the idea that NEXMIF pathology is functional rather than structural. An unexpected finding was the increased body length observed in nexmifa/b mutant larvae. Although postnatal growth delay has been reported in some individuals with NEXMIF-related disorders, this phenotype was assessed at an early larval stage and may therefore not directly reflect postnatal growth in humans. Future studies will be required to determine whether this early developmental phenotype persists or normalizes during later stages of development.

At the molecular level, NEXMIF regulates N-cadherin/β1-integrin–dependent adhesion complexes that drive early synapse assembly and neurite extension (Gilbert and Man, 2016; Magome et al., 2013). Consistently, our RNA-seq data show strong enrichment for cell-adhesion terms alongside MAPK signaling—a pathway linking adhesive signals to activity-dependent synaptic maturation (Iroegbu et al., 2021; Tepass et al., 2000). We also observed dysregulation of sodium-ion transport genes, a category that includes several DEE driver channels and transporters whose malfunction amplifies network excitability (Brunklaus et al., 2020; Wang et al., 2011). The simultaneous disruption of cadherin–integrin scaffolds, MAPK relays and sodium-handling machinery places NEXMIF at a convergent node that integrates structural adhesion with electrical stability—precisely the two functional axes most often compromised in genes linked to ID and epilepsy.

Upregulation of *entpd3* and *ddx43* may represent a putative compensatory response associated with the hyperlocomotor phenotype. *ENTPD3*, a purinergic ectonucleotidase enriched in sensory and hypothalamic neurons (Vongtau et al., 2011; Appelbaum et al., 2007), degrades extracellular ATP and limits the excitatory drive that exacerbates seizures (Beamer et al., 2021). *DDX43*, a DEAD-box helicase linked to neuropsychiatric disorders and activity-dependent transcriptome remodeling (Wockner et al., 2015), maintains RNA-processing homeostasis. Only simultaneous genetic targeting of both genes exacerbated the hyperlocomotor phenotype, suggesting that ENTPD3 and DDX43 may act through a shared compensatory response. However, the molecular mechanisms underlying this interaction remain to be determined.

Conversely, *kbtbd7,* which encodes an E3-ubiquitin-ligase adaptor, was downregulated in *nexmifa/b* mutants, and further CRISPR/Cas9-mediated targeting of *kbtbd7* exacerbated the hyperlocomotor phenotype. These findings suggest that reduced Kbtbd7 activity may contribute to phenotype severity, although the underlying mechanism remains to be determined. Reduced *Kbtbd7* levels permit heightened NF-κB signaling and increased release of pro-inflammatory cytokines such as IL-1β and TNF-α; these mediators elevate neuronal excitability and lower the threshold for ictal activity (Shen et al., 2024; Vezzani and Viviani, 2015). These findings suggest that reduced Kbtbd7 activity may contribute to phenotype severity in the *nexmifa/b* mutant model. Future work should assess whether restoration of *kbtbd7* expression, for example, by overexpression approaches, can mitigate the behavioral phenotype and clarify its role as a disease modifier. To our knowledge, *entpd3, ddx43*, and *kbtbd7* have not previously been established as NEXMIF-associated biomarkers or modifiers in patients or experimental models of NEXMIF deficiency. We therefore interpret these genes as candidate modulators emerging from our zebrafish transcriptomic and functional analyses, whose conservation and relevance in mammalian models and patient-derived datasets will require future investigation.

Drug discovery for rare diseases is constrained by small patient numbers and high trial drop-out rates; repurposing or natural-product libraries provide accelerated pathways (Tambuyzer et al., 2020). Our 87-compound screen identified rauwolscine, veratridine and kahweol acetate as potent *entpd3* inducers. None, however, elevated mRNA levels of *ddx43*, and the only candidate advanced to behavioral testing, rauwolscine, failed to normalize the hyperlocomotion phenotype. This outcome underscores that *entpd3* upregulation alone is insufficient to predict behavioral rescue and suggests that effective rescue may require broader molecular modulation beyond entpd3 alone, potentially including *ddx43* or additional pathway markers. Similar limitations are seen in PCDH19 epilepsy—another X-linked DEE driven by cellular interference—where conventional single-pathway treatments such as clobazam rarely achieve complete seizure control (Dibbens et al., 2008; Lotte et al., 2016).

The pharmacological profiles of kahweol acetate, veratridine, and rauwolscine further clarify this requirement. Kahweol acetate, a coffee diterpene that activates PI3K/P38-NRF2 signaling, protects dopaminergic neurons against oxidative stress and improves functional outcome after traumatic brain injury (Hwang and Jeong, 2008; Lee et al., 2019). Veratridine provokes membrane-potential oscillations specific to the epilepsy-linked NaV1.7 channel, driving a marked rise in *entpd3* post-exposure (Lummis et al., 2025). A recent review on adrenergic signaling in epilepsy describes high-affinity α_2_-antagonists such as rauwolscine as context-dependent modulators of seizure threshold (Sitnikova et al., 2023), which may help explain why isolated *entpd3* induction failed to rescue behavior in our larvae.

Based on these findings, we propose a two-step screening pipeline: (1) a primary molecular screen that selects compounds upregulating *entpd3* and (2) secondary validation of *ddx43* mRNA upregulation*.* In this proposed strategy, candidates modulating both molecular targets would then proceed to behavioral testing. Such multi-parameter pipelines align with systems-biology principles: even monogenic rare diseases often evolve into complex, multi-gene network disturbances best addressed with multi-target pharmacology (Pushpakom et al., 2019; Uffelmann et al., 2021). This staged approach conserves resources, avoids unnecessary downstream assays, and increases the probability of identifying compounds with genuine disease-modifying potential.

Some limitations should be acknowledged. First, the F0 CRISPR/Cas9 functional assays represent mosaic genomic perturbations rather than complete gene inactivation. Thus, the effects observed after targeting *entpd3*, *ddx43*, and *kbtbd7* should be interpreted as functional evidence supporting candidate modifier activity, rather than as definitive proof of complete loss of function. This is particularly relevant for *kbtbd7*, where the modest editing efficiency may underestimate the effect of stronger disruption. In addition, the *entpd3*-based screen was designed as a scalable first-pass prioritization strategy, rather than as a standalone predictor of behavioral rescue, as illustrated by the rauwolscine results. Another important consideration is that, although simultaneous disruption of *nexmifa* and *nexmifb* reduces overall Nexmif activity and provides a useful vertebrate loss-of-function model, zebrafish larvae do not reproduce X-linked inheritance, X-chromosome-inactivation-driven mosaicism, or the sex-specific effects observed in human patients. Therefore, this model should be interpreted as a tractable system to study selected consequences of reduced Nexmif activity and to prioritize candidate modifiers, rather than as a complete representation of all clinical features of NEXMIF-related encephalopathy.

In summary, this study establishes a stable double *nexmifa/b* loss-of-function zebrafish model to study selected features of NEXMIF-related encephalopathy. By integrating behavioral phenotyping, transcriptomic profiling, and F0 CRISPR-mediated functional perturbation, we identified *entpd3*, *ddx43*, and *kbtbd7* as candidate modulators of the hyperlocomotor phenotype. Our findings support a working model in which increased *entpd3* and *ddx43* expression may reflect a compensatory response, whereas reduced *kbtbd7* activity may contribute to phenotype severity (Figure 8). Although these hypotheses require further mechanistic and cross-species validation, the *nexmifa/b* model provides a tractable vertebrate platform for investigating disease-relevant pathways and prioritizing therapeutic strategies for XLID-98.

**FIGURE 8:**
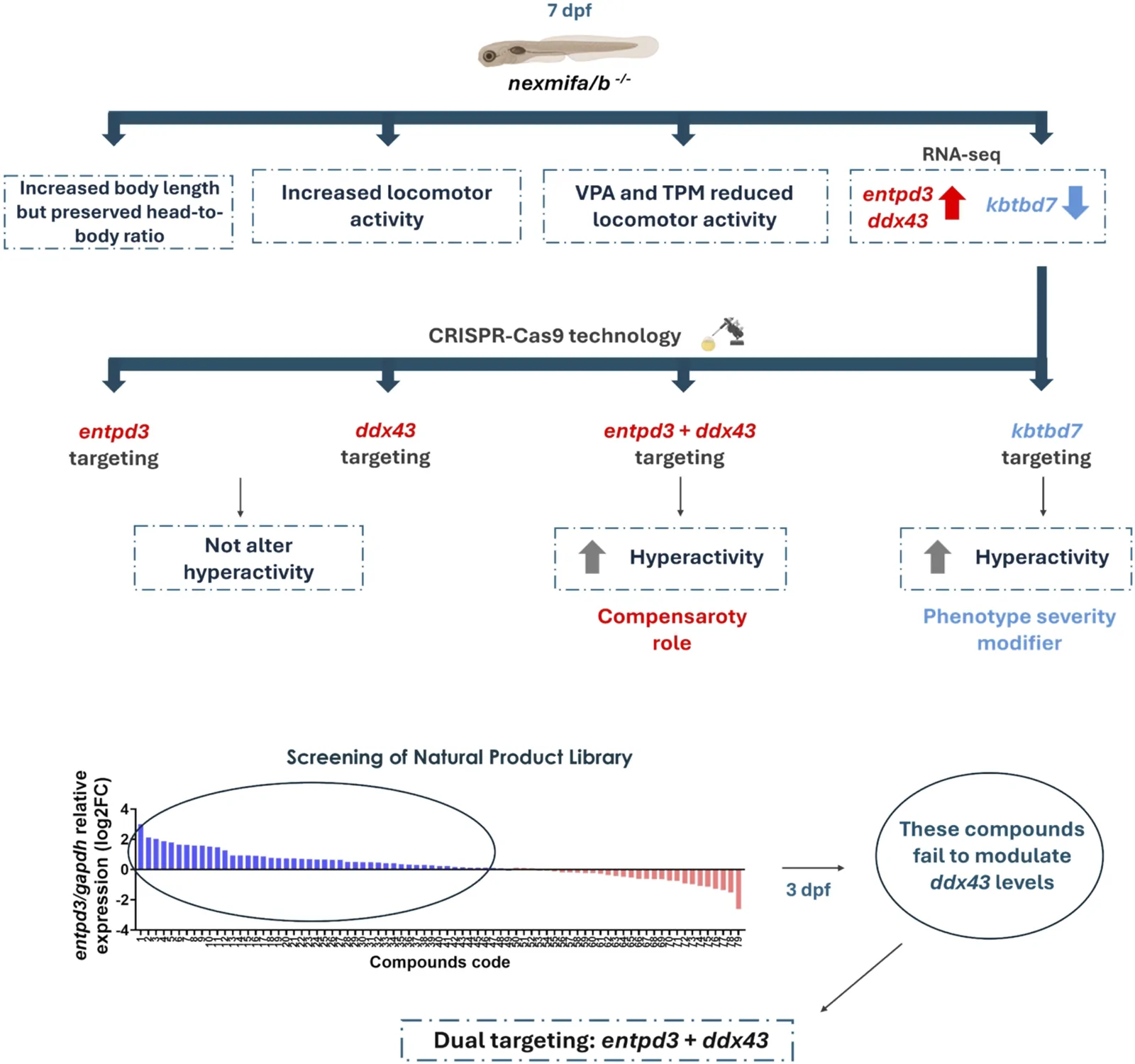
Schematic summary. At seven dpf, *nexmifa/b* ^−/−^ larvae display marked morphological alterations, characterized by an increased total body length while preserving a normal head-to-body ratio. Mutant larvae also exhibit increased locomotor activity, which is significantly reduced by treatment with valproic acid (VPA) or topiramate (TPM). Transcriptomic analysis of larval heads at seven dpf identified 414 differentially expressed genes (DEGs), from which *entpd3* and *ddx43* were selected as upregulated candidates, and *kbtbd7* as a downregulated gene. Individual genetic disruption of *entpd3* or *ddx43* did not modify the hyperlocomotor phenotype, whereas their combined disruption exacerbated hyperactivity, supporting a putative compensatory interaction between these genes. In parallel, further disruption of *kbtbd7* increased hyperactivity, suggesting that reduced *kbtbd7* mRNA levels may contribute to phenotype severity. A total of 87 compounds from the SCREEN-WELL® Natural Product Library were tested, of which 49 compounds (blue bars) further increased *entpd3* transcript levels without affecting *ddx43* mRNA levels, highlighting the need for broader molecular validation beyond a single-gene readout.

## Materials and methods

### Animals

Zebrafish (*Danio rerio* H.) were obtained from the Zebrafish International Resource Center and mated, staged, raised, and processed as described (Westerfield, 1993).

### CRISPR-Cas9 injections in zebrafish

The CRISPR RNA (crRNA) for zebrafish *nexmifa*, *nexmifb*, *entpd3*, *ddx43*, *kbtbd7* (Supplementary Table S1), negative control crRNA (Catalog no. 1072544, crSTD), and trans-activating CRISPR RNA (tracrRNA) were obtained from Integrated DNA Technologies (IDT). They were resuspended in duplex buffer at 100 μM, and 1 μL was incubated with 1 μL of 100 μM tracrRNA at 95 °C for 5 min and then 5 min at room temperature to form the complex. Then, the crRNA-tracrRNA complex was diluted with 1.45 μL of duplex buffer. For the final injection mix, 1 μL of this complex was combined with 0.25 μL of Cas9 Nuclease V3 (IDT, 1081058) (10 mg/mL), 0.25 μL of phenol red, and 2.55 μL of duplex buffer. When two crRNAs were co-injected, each was first annealed separately with tracrRNA, and then 1 μL of each duplex was mixed with 0.30 μL of Cas9 Nuclease V3 (10 mg/mL), 0.25 μL of phenol red, and 1.55 μL of duplex buffer. Throughout this work, the term guide RNA (gRNA) refers to the annealed crRNA–tracrRNA duplex. The final mix was injected into the yolk sac of one-to eight-cell-stage embryos using a microinjector (Narishige) (0.5–1 nL per embryo). The same amounts of gRNA were used in all experimental groups. The efficiency of each gRNA was tested by amplifying the target sequence with a specific pair of primers (Supplementary Table S2). The resulting amplicon was sequenced by Sanger sequencing and analyzed using the ICE webtool webtool from Synthego (https://ice.synthego.com/#/) to determine the indel rate and knockout score, which estimates the proportion of edited sequences carrying disruptive indels, particularly those that are not multiples of three and are therefore predicted to generate frameshifts, premature stop codons, and truncated proteins (Conant et al., 2022).

### Morphological assessment

At 7 days post fertilization (7 dpf), *nexmifa/b* ^+/+^ and *nexmifa/b* ^−/−^ larvae were maintained at 28.5 °C under 14 h: 10 h light-dark cycle in egg water (60 mg/L sea salts in distilled water). Larvae were anesthetized in egg water with 0.16 mg/mL tricaine and imaged in bright field using a Leica MZ16FA fluorescence stereomicroscope. Total body length was measured as the straight-line distance from the anterior tip of the head to the distal margin of the caudal fin, whereas head size was determined by outlining the region extending from the same anterior point to the junction between the head and trunk. For each larva, the head-to-body-length ratio was calculated using Fiji/ImageJ. Image calibration was performed using the scale bar included in each image, allowing conversion from pixels to micrometers before quantification.

### Behavioral assays

At six dpf, larvae were placed in 48-well plates (three larvae per well, 300 μL egg water) and habituated overnight at 28.5 °C under 14 h: 10 h light–dark cycle. This configuration, which minimizes compound usage, was applied uniformly across all groups, including untreated groups. At seven dpf, larvae were transferred individually to a 96-well plate (one larva per well) containing 200 μL of egg water and placed in a DanioVision Observation Chamber (Noldus Information Technology) maintained at 28.5 °C. Locomotor activity was assessed using the Visual-Startle OFF protocol, adapted from (Weinschutz Mendes et al. (2023)). After a 60-min acclimation period under constant white light, larvae were exposed to five 1-s dark pulses separated by 29 s of light. Behavioral tracking was performed at 30 frames per second using EthoVision XT software (version 15). The following parameters were quantified: total distance moved (mm), movement duration (s), and maximum acceleration (mm/s^2^). Measurements included the final 29 s of the acclimation period and each stimulus response. Behavioral acquisition was performed using the automated DanioVision system with predefined tracking and analysis parameters. Because of the breeding and treatment design, full blinding to genotype and treatment group was not feasible in all experiments. However, automated acquisition and standardized analysis settings minimize observer-dependent bias.

### Chemical treatments of zebrafish larvae

For behavioral assays, six dpf larvae were placed in 48-well plates (three larvae per well) and treated by bath immersion with the following compounds diluted in egg water supplemented with 1% dimethyl sulfoxide (DMSO): 2.5 mM valproic acid sodium (VPA) (MCE, HY-10585A), 5 μM topiramate (TPM) (Selleckchem, S1438), or 5 μM rauwolscine hydrochloride (MCE, HY-12710). At seven dpf, larvae were transferred individually to a 96-well plate (one larva per well) for behavioral analysis.

For gene expression analyses, 1-dpf embryos were manually dechorionated and incubated for 48 h at 28.5 °C with 87 compounds of the SCREEN-WELL® Natural Product library (ENZO, BML2865) diluted in egg water containing 1% DMSO. The following positive hits were validated at different concentrations in six-well plates with 25 larvae per well: kahweol acetate (MCE, HY-N6258), veratridine (MCE, HY-N6691) and rauwolscine hydrochloride (MCE, HY-12710).

### Gene expression analysis

For conventional two-step RT-qPCR, total RNA was extracted from pools of 25 whole larvae (3 dpf) or 40 larval heads (7 dpf) using TRIzol™ Reagent (Invitrogen) followed by purification with either RNAqueous™-Micro Total RNA Isolation Kit or PureLink™ RNA Mini Kit (both from Invitrogen), respectively, according to the manufacturer’s instructions. Total RNA was treated with deoxyribonuclease I DNase I, amplification grade (1 U/μg RNA, Invitrogen). SuperScript™ IV VILO cDNA Synthesis Kit (Invitrogen) was used to synthesize first-strand cDNA with random primer from 1 μg of total RNA. Real-time PCR was performed with a QuantStudio Real-Time PCR Systems instrument (ThermoFisher Scientific) using SYBR Green PCR Core Reagents (Applied Biosystems). Reaction mixtures were incubated for 10 min at 95 °C, followed by 40 cycles of 15 s at 95 °C, 1 min at 60 °C, and finally 15 s at 95 °C, 1 min 60 °C, and 15 s at 95 °C. For each mRNA, gene expression was normalized to the ribosomal protein S11 gene (*rps11*) content in each sample, using the Pfaffl method (Pfaffl, 2001). In all experiments, each PCR was performed in technical triplicate. A representative experiment of three independent biological replicates is shown in the figures. The primer sequences used are described in Supplementary Table S3.

For one-step RT-qPCR, Applied Biosystems™ TaqMan™ Gene Expression Assays were used in a duplex format and include optimized components that allow reverse transcription and PCR amplification to take place in the same reaction. Each assay contains an unlabeled primer pair, and a hydrolysis probe labeled at the 5′ end with either FAM or VIC and at the 3′ end with a minor-groove binder (MGB) and a non-fluorescent quencher (NFQ) (Thermo Fisher Scientific). Pools of five 3-dpf larvae were homogenized in 100 μL of lysis buffer (0.5× TBE, 1% Tween-20) using a motorized pestle. Homogenates were incubated at 90 °C for 10 min and centrifuged at maximum speed for 5 min. The resulting supernatant was diluted 1:5 with water, and this dilution was used directly as the template for RT-qPCR. Reactions were carried out in a 10 μL final volume containing 2.5 μL of 4× TaqMan™ One-Step Master Mix (Applied Biosystems), 0.5 μL of VIC-labeled *gapdh* assay (Assay ID: 11927021), 0.5 μL of FAM-labeled *entpd3* assay (Assay ID: 11977021), 5.6 μL of diluted lysate, and nuclease-free water to adjust the final volume. Reactions were run in a QuantStudio 5 Real-Time PCR System using the following thermal profile: 25 °C for 2 min, 53 °C for 10 min (reverse transcription), 95 °C for 2 min, followed by 40 cycles of 95 °C for 3 s and 60 °C for 30 s. All TaqMan™ probes spanned exon–exon junctions to avoid amplification of genomic DNA.

### RNA-sequencing

RNA-sequencing (RNA-seq) was performed in two groups of 7-dpf zebrafish larval heads—WT (*nexmifa/b* ^+/+^) and double knock-out (*nexmifa/b* ^−/−^). Each group consisted of three biological replicates, with 40 pooled heads per replicate. Total RNA was extracted with TRIzol™ Reagent (Invitrogen), and RNA quality, including yield, purity, and integrity, was assessed before library preparation. All RNA samples passed quality control, with RNA integrity values ranging from 9.8 to 9.9. Library preparation, Illumina sequencing, and primary bioinformatic processing were performed by Novogene (Zhang et al., 2017). DEGs were identified if they had a log_2_ fold change < −0.25 or >0.25 and adjusted *p*-value (q) < 0.05 (Thakur et al., 2014). A relatively permissive fold-change threshold was selected to increase sensitivity for detecting moderate transcriptional changes in pooled larval head samples, while maintaining statistical significance (adjusted p-value <0.05) as the primary criterion for DEG identification. Hierarchical clustering analysis was applied to the DEG sets. Gene Ontology (GO) and Kyoto Encyclopedia of Genes and Genomes (KEGG) pathway enrichment analyses were performed, and terms with corrected *p*-values <0.05 were considered significantly enriched.

### Statistical analysis

Data were shown as mean ± standard error of the mean (SEM). All statistical analyses were performed in GraphPad Prism v8.0. Data normality was evaluated with the Shapiro–Wilk test. For comparisons between two groups, normally distributed datasets were analyzed with an unpaired Student’s t-test (Welch’s correction applied when variances were unequal), whereas non-normal datasets were assessed with the Mann–Whitney *U* test. For comparisons involving more than two groups, normally distributed data were examined by one-way ANOVA followed by Tukey’s *post hoc* test (or Welch’s ANOVA when variances were unequal), and non-normal data were analyzed with the Kruskal–Wallis test.

## Data Availability

The RNA-seq data have been deposited in GEO under accession number GSE334219 and are publicly available.
